# Supplementary material for: Sequential phenotypic constraints on social information use in wild baboons
Source: eLife. 2016 Apr 12;5:e13125. doi: 10.7554/eLife.13125 (PMC4829417; doi:10.7554/eLife.13125)
Supplement: Supplementary file 1. — Shown are the combinations of individual-level variables, whether the model was multiplicative, additive or neither (when the individual-level variables were not included) (model type), whether individual-level variables were included (0 = no, 1 = yes); whether the process was modelled as a social or asocial diffusion (social/asocial); the AICc of the model; the difference from the AICc of the best model (∆AICc); the support for the model (model weight); and the relative weight of the model in comparison to the model set. DOI: http://dx.doi.org/10.7554/eLife.13125.017 [file elife-13125-supp1.docx]

**Supplementary file 1**

Comparisons of the different models used to assess the effect of individual-level variables on the transmission of information among individuals, with ∆AICc <2 considered to have good support.

| **individual-level variables** | **model type** | **social/asocial** | **AICc** | **∆AICc** | **model weight** | **relative weight** |
| --- | --- | --- | --- | --- | --- | --- |
| boldness rank age | Multiplicative | social | 3967.71 | 0.00 | 1.000 | 0.260 |
| rank age | Multiplicative | social | 3968.49 | 0.78 | 0.677 | 0.176 |
| boldness sex rank age | Multiplicative | social | 3968.90 | 1.19 | 0.552 | 0.144 |
| sex rank age | Multiplicative | social | 3969.00 | 1.29 | 0.525 | 0.137 |
| boldness sex rank | Multiplicative | social | 3969.16 | 1.44 | 0.487 | 0.127 |
| boldness rank | Multiplicative | social | 3970.09 | 2.37 | 0.306 | 0.080 |
| sex rank | Multiplicative | social | 3971.20 | 3.49 | 0.175 | 0.045 |
| sex | Multiplicative | social | 3974.22 | 6.51 | 0.039 | 0.010 |
| boldness sex | Multiplicative | social | 3975.25 | 7.54 | 0.023 | 0.006 |
| sex age | Multiplicative | social | 3975.30 | 7.59 | 0.022 | 0.006 |
| rank | Multiplicative | social | 3976.46 | 8.74 | 0.013 | 0.003 |
| boldness sex age | Multiplicative | social | 3976.90 | 9.18 | 0.010 | 0.003 |
| boldness rank age | Additive | social | 3979.15 | 11.44 | 0.003 | 0.001 |
| boldness sex rank | Additive | social | 3979.40 | 11.69 | 0.003 | 0.001 |
| boldness sex rank age | Additive | social | 3979.43 | 11.71 | 0.003 | 0.001 |
| boldness rank | Additive | social | 3981.52 | 13.81 | 0.001 | 0.000 |
| boldness age | Additive | social | 3981.56 | 13.85 | 0.001 | 0.000 |
| age | Multiplicative | social | 3982.53 | 14.82 | 0.001 | 0.000 |
| boldness sex age | Additive | social | 3983.58 | 15.87 | 0.000 | 0.000 |
| boldness age | Multiplicative | social | 3983.70 | 15.99 | 0.000 | 0.000 |
| boldness | Additive | social | 3984.07 | 16.36 | 0.000 | 0.000 |
| boldness | Multiplicative | social | 3985.01 | 17.30 | 0.000 | 0.000 |
| boldness sex | Additive | social | 3985.21 | 17.50 | 0.000 | 0.000 |
| rank | Additive | social | 3986.82 | 19.11 | 0.000 | 0.000 |
| 0 | NA | social | 3987.01 | 19.30 | 0.000 | 0.000 |
| sex rank | Additive | social | 3987.42 | 19.71 | 0.000 | 0.000 |
| age | Additive | social | 3988.07 | 20.36 | 0.000 | 0.000 |
| rank age | Additive | social | 3988.29 | 20.58 | 0.000 | 0.000 |
| sex | Additive | social | 3988.65 | 20.94 | 0.000 | 0.000 |
| sex rank age | Additive | social | 3989.41 | 21.70 | 0.000 | 0.000 |
| sex age | Additive | social | 3990.09 | 22.38 | 0.000 | 0.000 |
| boldness rank age | NA | asocial | 4160.02 | 192.31 | 0.000 | 0.000 |
| boldness sex rank age | NA | asocial | 4161.76 | 194.05 | 0.000 | 0.000 |
| rank age | NA | asocial | 4166.63 | 198.91 | 0.000 | 0.000 |
| sex rank age | NA | asocial | 4167.65 | 199.94 | 0.000 | 0.000 |
| boldness sex age | NA | asocial | 4170.59 | 202.87 | 0.000 | 0.000 |
| sex age | NA | asocial | 4172.85 | 205.14 | 0.000 | 0.000 |
| boldness sex rank | NA | asocial | 4173.40 | 205.69 | 0.000 | 0.000 |
| boldness age | NA | asocial | 4176.26 | 208.55 | 0.000 | 0.000 |
| boldness rank | NA | asocial | 4176.49 | 208.78 | 0.000 | 0.000 |
| boldness sex | NA | asocial | 4176.66 | 208.95 | 0.000 | 0.000 |
| age | NA | asocial | 4179.05 | 211.34 | 0.000 | 0.000 |
| sex | NA | asocial | 4188.11 | 220.39 | 0.000 | 0.000 |
| sex rank | NA | asocial | 4189.67 | 221.96 | 0.000 | 0.000 |
| boldness | NA | asocial | 4190.68 | 222.97 | 0.000 | 0.000 |
| rank | NA | asocial | 4203.93 | 236.22 | 0.000 | 0.000 |
| 0 | NA | asocial | 4210.16 | 242.45 | 0.000 | 0.000 |

Shown are the combinations of individual-level variables, whether the model was multiplicative, additive or neither (when the individual-level variables were not included) (model type), whether individual-level variables were included (0 = no, 1 = yes); whether the process was modelled as a social or asocial diffusion (social/asocial); the AICc of the model; the difference from the AICc of the best model (∆AICc); the support for the model (model weight); and the relative weight of the model in comparison to the model set.
